# Financial stress in emerging adults with type 1 diabetes: a mini review integrating lessons from cancer research

**DOI:** 10.3389/fcdhc.2024.1328444

**Published:** 2024-01-26

**Authors:** Katherine Wentzell, Kathryn E. Nagel

**Affiliations:** ^1^ Pediatric, Adolescent, and Young Adult Section, Joslin Diabetes Center, Boston, MA, United States; ^2^ Divisions of Endocrinology and Pediatric Endocrinology, Massachusetts General Hospital, Boston, MA, United States

**Keywords:** emerging adult, young adult, type 1 diabetes, financial toxicity, cancer

## Abstract

Amongst adults in the United States, those ages 18-30 have the highest unemployment rates, the lowest incomes, and are the most likely to be uninsured. Achieving financial independence is a core developmental task for this age group, but for those with type 1 diabetes (T1D), the high costs of insulin and diabetes supplies as well as an employment-based insurance model with minimal safety net can make this a formidable challenge. Cost-related non-adherence to diabetes management is particularly high in emerging adults with T1D and is associated with severe consequences, such as diabetic ketoacidosis (DKA) and even death. Objective financial burden and subjective financial distress related to illness are not unique to diabetes; in cancer care this construct is termed financial toxicity. Researchers have identified that emerging adults with cancer are particularly vulnerable to financial toxicity. Such research has helped inform models of care for cancer patients to mitigate cost-related stress. This mini review aims to briefly describe the state of the science on financial stress for emerging adults with T1D and explore parallels in cancer scholarship that can help guide future work in diabetes care to reduce health inequity, drive research forward, improve clinical care, and inform policy debates.

## Financial burden in emerging adults with T1D

Emerging adulthood is a complex developmental period between 18 and 30 years of age marked by numerous transitions and a high degree of uncertainty (1). A core task of this developmental stage is achieving financial independence from childhood caregivers (2). For people living with type 1 diabetes (T1D), achieving financial independence can be particularly daunting given the high costs of insulin and other diabetes supplies and, in the United States, an employment-based insurance model with minimal safety net (3). As insulin prices have risen exponentially over the past 20 years (4, 5), catastrophic spending, defined as using more than 40% of one’s income for medical supplies, has affected 14% of Americans with diabetes (6). As many as 1 in 4 patients have rationed insulin due to cost (7). The most extreme cases of cost-related non-adherence (CRN) have resulted in death (8, 9). These devastating consequences are disproportionately affecting young adults (10), who lack the financial and employment security that is characteristic of later stages of life.

All those ages 18 to 30 have the highest rate of unemployment (11), the lowest incomes (12), and are most likely to be uninsured (13). Furthermore, there is higher job turnover during this time of life (14), which in the employment-based insurance structure in the United States, requires frequent switching between insurance carriers. Each insurance transition can be associated with barriers in obtaining diabetes supplies as well as possible lapses in coverage. For many young adults, turning 26 years old is a particularly stressful hurdle as federal law mandates obtaining health insurance independent of parents by this age (15). Emerging adults must also transition from pediatric to adult diabetes care, which often necessitates leaving the providers and care teams they have grown to know and trust. Such transitions are associated with further risk of becoming disconnected from the healthcare system and seeking care in suboptimal settings such as emergency rooms (16, 17).

Unsurprisingly, given these dynamics and the increasingly expensive nature of diabetes care, recent small sample surveys of young adults with T1D have found a prevalence of CRN as high as 40 to 60% (18, 19). In addition to paying for insulin, individuals with T1D also face other health-related costs including doctors’ visits, supplies (including diabetes technologies), medications for comorbid conditions, and the loss of income and/or educational opportunity to attend to the multiple management needs of diabetes (20).

While the full extent to which CRN may be affecting outcomes in emerging adults with T1D needs further investigation, data show that in the United States this age group has a mean A1c of 8.9% (21) with only 12% of emerging adults meeting recommended glycemic targets (22). Financial stress has been linked to reductions in self-management behaviors in emerging adults with T1D (3). Less frequent self-management behaviors are correlated with worse glycemic outcomes (23) and may contribute to the recent observed trends of suboptimal glycemic control in this population.

Suboptimal glycemic control increases the risk for microvascular and macrovascular complications (24). The implications of developing complications for young people, who ideally have many decades of life ahead of them, are considerable. Furthermore, young adulthood encompasses prime childbearing years. Suboptimal glycemic control during gestation is known to increase the risk of maternal and fetal complications including congenital malformations (25). Given the far-reaching impacts of suboptimal glycemic control during this time of life, it is important to identify targetable factors, such as financial stress, that may contribute to these trends.

Recent qualitative work has explored the impact of financial burden on the lived experience of emerging adults with T1D. These studies identify that emerging adults carry substantial emotional distress related to managing diabetes costs (26–28). A recent report showed that 90% of emerging adult participants expressed worry related to the costs of their diabetes. In open-ended responses, they characterized their concern using words such as ‘fear,’ ‘overwhelm,’ ‘isolation,’ and, ‘limitation’ (26). In another report, participants explained that the challenges in affording diabetes supplies can delay age-appropriate maturation and financial independence, as they must rely on their parents for financial support for longer than their peers without diabetes (27). Several of the participants in this study expressed guilt as they realized how much their disease had been costing their parents (27).

A recurring theme in these qualitative studies is frustration at institutional indifference to the barriers faced trying to obtain diabetes care and supplies (26–28). Participants identified insurance hurdles and poor communication between different players in the healthcare system as contributing factors in lapses in filling prescriptions (27). In an attempt to reduce out-of-pocket costs, some individuals turn to an underground market or online fundraising to obtain their diabetes supplies (29, 30). Underground markets have been associated with safety concerns such as improperly stored insulin or dysfunctional medical equipment (29). Crowd sourcing requires public online disclosure of personal information, such as health status, and carries the risk of exploitation (30). Emerging adults appear to experience substantial financial distress and, in the absence of an adequate safety net, they can turn to risky problem solving behaviors to avoid hospitalization or even death.

## Lessons from cancer care

The impact of disease-related financial burden is only starting to be understood in diabetes care; research on this topic is lacking and interventions are not a routine part of clinical care. However, in cancer care, this topic has been more thoroughly researched and a construct describing how people are negatively impacted by the cost of healthcare, termed financial toxicity, has been developed (31). The construct of financial toxicity has two components: objective financial burden and subjective financial distress. Objective financial burden is the ratio of total out-of-pocket spending on health-related costs compared to income. Subjective financial distress is the affective experience of financial burden and includes feelings of worry or anxiety about current and/or future costs of managing health. In cancer care, financial toxicity is a recognized side effect of cancer treatment (32) and multiple professional organizations recommend assessing for financial toxicity during acute treatment and follow-up care (33).

Though this construct has been studied extensively across the lifespan in those living with cancer, emerging adults have been identified as uniquely vulnerable to financial toxicity (34, 35). Emerging adults typically have not yet achieved financial independence from their family of origin or accumulated enough savings to weather a financial shock, such as cancer treatment. Emerging adults living with cancer can also experience interruptions in education and career trajectories as they pursue treatment (36). Importantly, the experience of financial toxicity as an emerging adult with cancer is associated with worse mental health (37), poor adherence to cancer treatment (38), and increased mortality (39). The impact of financial toxicity during emerging adulthood has lifelong sequelae, including continued high levels of financial toxicity for many years after a cancer diagnosis (40) and increased risk of bankruptcy (41) throughout the duration of survivorship.

## Future directions

Recently, researchers have begun to make the connection between financial toxicity in cancer to the experience of cost-related burden in people living with diabetes (42). These parallels may be even more salient in the emerging adult population (43). Much of what occurs during this time of life can have long-term effects on the trajectory of one’s career, family planning, and financial security. When illness strikes at this age, normal developmental tasks can be sidelined and future planning threatened. Especially with the current healthcare infrastructure of the United States, which provides little security in times of transition, the financial toll of disease at this time of life can have severe and long-lasting consequences.

In cancer care, financial toxicity has also been recognized as a driver of health inequity (44). Research on this topic in cancer has helped drive innovations to mitigate these concerns such as routine screening for financial toxicity, increased communication about cost of care during healthcare visits, and staffing models that include adequate social work and patient navigator support (45, 46). The construct of financial toxicity has also helped with articulating calls to action for policy changes that can lower costs such as Medicare negotiation of drug pricing, value-based pricing models, and enhanced roles of regulatory agencies on the drug market (45, 46).

Diabetes has gained increasing attention for unattainably high costs of care (4). Using the model of financial toxicity can help streamline research efforts to better understand the extent of this problem. Identifying financial toxicity as a complication of diabetes can help direct efforts, just as they have in cancer care. Regular screening for financial toxicity and normalizing communication on this topic by the diabetes care team should be considered and studied for effectiveness. Recent work has adapted and validated a measure of financial toxicity used in cancer care to diabetes care by changing the word “cancer” to “diabetes in 1 item (42). Further work is needed to explore if this measure captures all facets of the experience of financial toxicity in diabetes management and to understand its utility as a screening measure. Supportive staffing models that give patients ready access to social workers and case managers who can help navigate financial and insurance challenges may also provide needed relief.

Cancer and diabetes diagnoses share parallels as life-threatening diseases that can strike at any age. Cancer often requires an intense period of very expensive treatment, whereas diabetes typically requires lifelong adherence to a chronic regimen. High costs of care give both diseases the potential to be financially devastating, particularly to young people who may not have accumulated savings and are trying to establish careers, potentially without or with limited access to reliable and affordable health insurance. Recognizing and quantifying this problem across disease states, using the construct of financial toxicity, can help inform clinical models of care and advise policy reform.

Insulin pricing has received substantial attention in recent U.S. policy debates. It is becoming increasingly recognized that costs of care for diabetes treatment are unattainable, a reality which has led to bipartisan public outcry (4). Several policy solutions have been proposed, and a few, such as state co-pay caps for insulin and the recent Inflation Reduction Act (IRA) (which allows Medicare negotiation on certain drugs including several for diabetes and implemented a $35 copay cap on insulin for Medicare recipients) have started to address affordability concerns for diabetes care. These solutions, however, are limited in scope most notably in that they require patients to have insurance coverage, and for the IRA specifically, apply only to those on Medicare who (by definition) are over 65 years old (47). Recognizing the unique financial burden diabetes places on young people is critical as further policy solutions are considered. Such efforts should focus on attainable drug pricing regardless of insurance status and affordable and comprehensive health insurance options that are not linked to employment status.

By embedding research, clinical care, and policy recommendations within the developmental stage of emerging adulthood and by integrating the well-formed concept of financial toxicity and the knowledge gained from research on this topic in cancer into diabetes care, future work can help improve health outcomes and mitigate distress for emerging adults living with T1D.

## Author contributions

KW: Conceptualization, Writing – original draft, Writing – review & editing. KN: Conceptualization, Writing – original draft, Writing – review & editing.
